# Color Stability of Resin Composites Immersed for Different Durations in Alcohol-Based and Alcohol-Free Mouthwashes: An In Vitro Study

**DOI:** 10.4317/jced.63174

**Published:** 2025-10-01

**Authors:** Jano Álvarez-Horna, Ana Aliaga-Mariñas, Leonor Castro-Ramirez, Carlos López-Gurreonero, Alberto Cornejo-Pinto, Rafael Scipión-Castro, César Cayo-Rojas

**Affiliations:** 1 culty of Dentistry, Universidad Nacional Federico Villarreal, Lima, Peru

## Abstract

**Background:**

Alcohol in some mouthwashes can dissolve the polymer chain of resin composites, potentially altering their properties. The aim was to evaluate the color stability of resin composites immersed for 24 hours and 7 days in alcohol-based and alcohol-free mouthwashes.

**Material and Methods:**

This experimental, longitudinal, *in vitro* study included 90 resin composite discs divided into three equal groups (n = 30): Filtek Z350XT, Tetric N-Ceram, and Opallis. Each group was split into two equal subgroups (n = 15) and immersed in Listerine Zero and Listerine Cool Mint. Color variation was recorded with a Vita Easyshade spectrophotometer after 24 hours and 7 days of immersion. Welch’s robust ANOVA with an intergroup factor and Student’s t-test for related samples were used, with significance set at *p*<0.05.

**Results:**

There was no significant color variation when comparing the effect of alcohol-based and alcohol-free mouthwashes on each resin composite (Opallis, Tetric N-Ceram, and Filtek Z350XT) at both 24 hours (*p*>0.05) and 7 days (*p*>0.05), although the average color variation in all samples exceeded the clinically accepTable threshold (ΔE > 3.3). When comparing resins immersed in alcohol-based mouthwash, Filtek Z350XT showed greater color variation at 24 hours (*p*<0.05) and 7 days (*p*<0.05). Tetric N-Ceram resin immersed in alcohol-based mouthwash significantly increased color variation (*p* = 0.036) between 24 hours and 7 days.

**Conclusions:**

In this *in vitro* study, all evaluated resin composites showed color variation exceeding the clinically accepTable threshold. Filtek Z350XT was the most susceptible to color change following exposure to alcohol-based mouthwashes, while Tetric N-Ceram showed a significant increase in color variation between 24 hours and 7 days. These findings suggest that the presence of alcohol in mouthwashes could negatively affect the color stability of certain resin composites.

** Key words:**Nanohybrid composite, comparative study, dental materials, resin composite, mouthwashes.

## Introduction

Resin composites are among the most widely used dental restorative materials for direct restorations, adhering to minimally invasive principles and offering an aesthetically pleasing appearance [1,2]. The classification of resin composites has primarily focused on filler size distribution, content, or composition, resulting in macrohybrid, microhybrid, nanohybrid, and hybrid resins [3,4]. Nanohybrid resin composites, which contain only nanoscale particles, offer improved polishability, reduced shrinkage, and superior aesthetics [4,5].

As patient expectations have evolved, it has been reported that the success of resin composite restorations depends not only on the restoration of masticatory function but also on color stability [6]. Color changes and marginal staining are among the most common causes of failure in resin composite restorations, leading to patient dissatisfaction and additional costs for replacement [6,7].

Color changes can result from intrinsic factors such as the photoinitiator system, matrix composition, filler load, particle size distribution, polymerization duration, and matrix monomer conversion, as well as extrinsic factors like staining beverages and habitual use of mouthwashes [2,7-9]. Mouthwashes have been widely used to prevent caries and periodontal diseases, and are considered a valuable adjunct to mechanical methods for controlling plaque, gingivitis, halitosis, and maintaining oral health [10-12]. However, the regular use of alcohol-based mouthwashes has raised concerns about their potential to accelerate the discoloration of resin composites, compromising esthetic outcomes over time [12,13].

Generally, mouthwash formulations consist of water, antimicrobial agents, salts, preservatives, and alcohol in varying concentrations [10,14,15]. However, it should be noted that alcohol acts as a solvent for the polymer chain of resin composites by removing monomers, oligomers, and linear polymers from the polymeric structure, which softens the resin surface and reduces its mechanical properties [10,11,14,15].

Chlorhexidine is one of the most studied mouthwashes in relation to resin composite color change [16-18]. However, there is limited evidence comparing the effects of alcohol-based and alcohol-free mouthwashes on the color stability of nanohybrid resin composites specifically. Most previous studies have focused on properties such as surface hardness or microleakage, with few addressing esthetic degradation using standardized colorimetric methods [10-12].

The aim of the study was to evaluate the color stability of three resin composites immersed for 24 hours and 7 days in alcohol-based and alcohol-free mouthwashes. The null hypothesis was that there are no significant differences in the color stability of resin composites immersed for 24 hours and 7 days in alcohol-based and alcohol-free mouthwashes.

## Material and Methods

1. Study Design

This experimental *in vitro* study was conducted at the Faculty of Dentistry of the National University Federico Villareal (UNFV) and in the Dent Import laboratory in Lima, Peru, between October and November 2024. It was approved by the Research Ethics Committee of the Faculty of Dentistry at UNFV with act No. 190-09-2024 dated October 7, 2024. Additionally, this study adhered to the CRIS guideline (Checklist for Reporting In-vitro Studies) [19].

2. Sample Size

**Figure 1:**
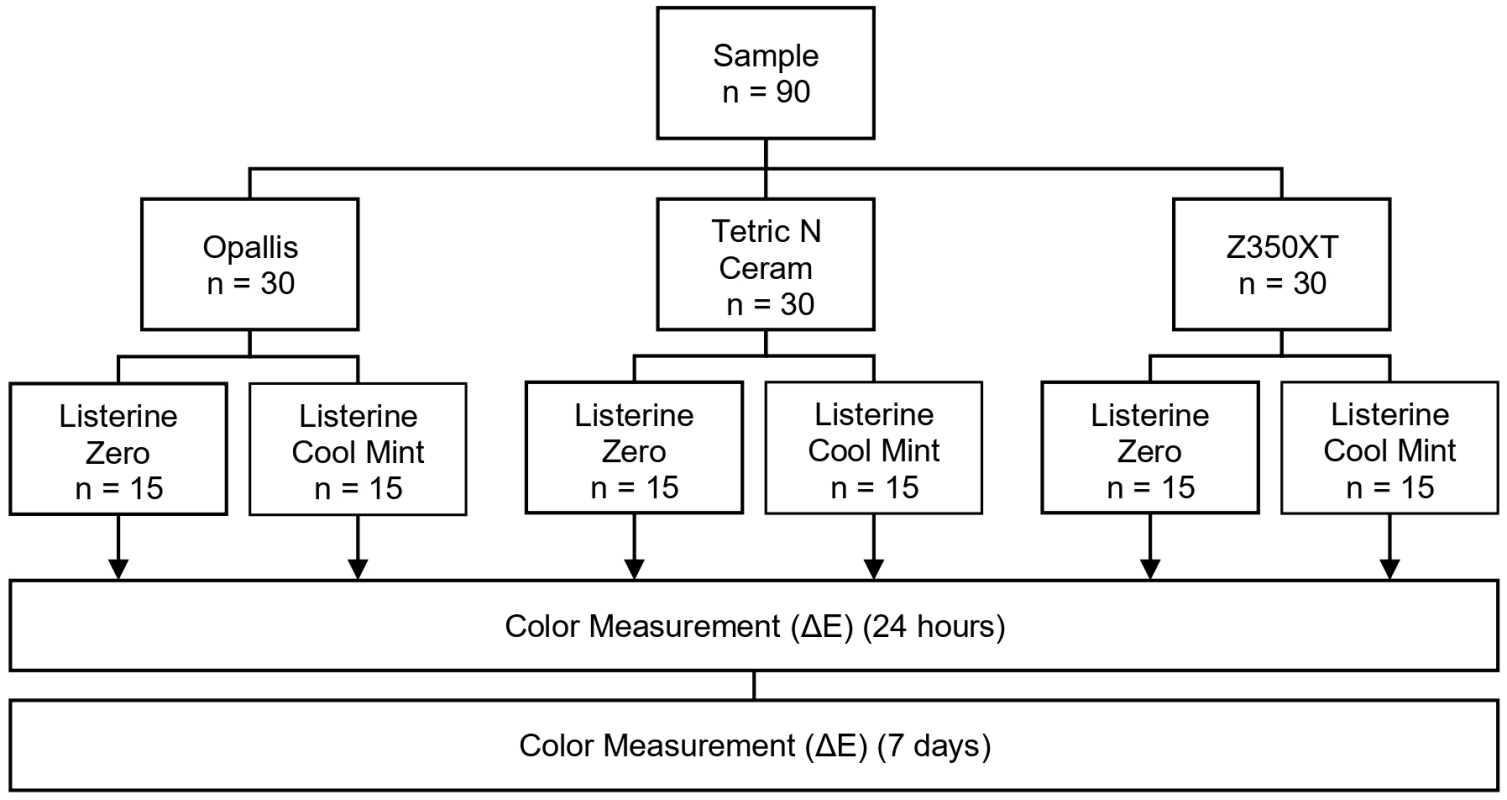
Random Distribution of Groups According to Sample Size.

Using the repeated measures ANOVA procedure in G*Power (version 3.1.9.7), the minimum required sample size (n = 90) was estimated based on data from a prior pilot study involving five sample units per group. For this calculation, a significance level (α) of 0.05 and a statistical power (1−β) of 0.80 were applied. An effect size of 0.338 was determined, considering six groups and two repeated measurements. Resin composite discs were randomly assigned into groups of fifteen and then immersed in either alcohol-based or alcohol-free mouthwash (Fig. 1).

3. Sample Preparation

Three resin composite materials were used: Opallis A2 (FGM, Santa Catarina, Brazil), Tetric® N-Ceram A2 (Ivoclar Vivadent, Schaan, Liechtenstein), and Filtek Z350XT A2 (3M ESPE, St. Paul, MN, USA) ( Table 1). A total of 30 discs were fabricated from each material, resulting in 90 specimens. All samples measured 8 mm in diameter and 2 mm in thickness, and were prepared by a single operator.

A standardized mold was employed for disc fabrication. Once the material was placed into the mold, celluloid strips were applied to both surfaces and gently pressed with a glass slide to remove any excess material [3,9,16]. Polymerization was performed using an LED curing unit (Woodpecker® LM-1, Woodpecker, Guilin, Guangxi, China) with an intensity of 1200 mW/cm² for 20 seconds. The light intensity was verified with a radiometer (Woodpecker® LM-1, Woodpecker, Guilin, Guangxi, China).

Each disc was then polished for 20 seconds using a contra-angle handpiece (NSK, Tokyo, Japan) connected to an electric motor, employing a four-step disc system (Sof-Lex, 3M ESPE) at 15,000 rpm. Polishing was performed by the same operator to ensure consistency. Afterward, all specimens were stored in sealed containers filled with distilled water at 37 °C for 24 hours to prevent external factors from influencing color measurements prior to the initial assessment [3,9,16].

Inclusion Criteria

– Nanohybrid resin composite discs of Opallis, Tetric N-Ceram, and Filtek Z350XT.

– Shade A2.

– Standardized dimensions: 8 mm in diameter × 2 mm in thickness.

Exclusion Criteria

– Discs with surface irregularities, internal bubbles, or signs of incomplete polymerization.

– Discs damaged during handling.

4. Immersion in Mouthwashes

Fifteen discs of each resin composite were immersed in 20 mL of Listerine Cool Mint (alcohol-based; Johnson & Johnson Healthcare Products) and 20 mL of Listerine Zero (alcohol-free; Johnson & Johnson Healthcare Products) [11,16]. The mouthwashes were replaced daily, and the temperature was consistently maintained at 37 °C during each renewal. All containers were kept sealed throughout the experiment to prevent fluid evaporation. Immersion periods were set at 24 hours and 7 days [11,12,17,20] ( Table 2).

5. Color Measurement

Prior to the immersion procedure, the baseline color of each sample was measured using a spectrophotometer (Vita Easyshade®, V Zahnfabrik, Bad Säckingen, Germany), following the ISO/Commission Internationale de l’Éclairage (CIE) 11664-6:2022 CIELAB standard [21].

The color parameters L*, a*, and b* were recorded, corresponding to lightness, red/green, and yellow/blue axes, respectively. Each sample was measured twice, and the spectrophotometer was calibrated according to the manufacturer’s instructions before each set of measurements. To ensure accurate readings, the probe tip was positioned perpendicularly and in contact with the sample surface. All measurements were performed inside a black box under standardized conditions of positioning, angulation, and ambient light.

**Figure 2:**
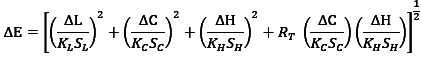
Formula.

After immersion in the mouthwashes for 24 hours and 7 days, the discs were rinsed with distilled water and dried with absorbent paper prior to color evaluation. All measurements were conducted by the same operator under identical conditions. Color change (ΔE) was calculated using the CIEDE2000 formula as follows: (Fig. 2).

Where ΔL, ΔC, and ΔH represent the differences in lightness, chroma, and hue between the initial and subsequent color measurements, respectively.

6. Statistical Analysis

All data were imported into SPSS (Statistical Package for the Social Sciences, version 28.0; IBM Corp., Armonk, NY, USA). Descriptive statistics included measures of central tendency and dispersion, such as the mean and standard deviation. For inferential analysis, normality was assessed using the Shapiro–Wilk test, and homogeneity of variances was evaluated using Levene’s test. Based on these preliminary assessments, parametric tests were selected. The Student’s t-test was used to compare either independent samples or paired measures, as appropriate. Additionally, Welch’s ANOVA was applied to compare three groups at two different time points. A *p-value* of <0.05 was considered statistically significant.

## Results

The color variability in the resin composites Opallis, Tetric N-Ceram, and Filtek Z350XT was similar, regardless of whether they were immersed for 24 hours in alcohol-based or alcohol-free mouthwashes (*p* = 0.219, *p* = 0.210, and *p* = 0.197, respectively). Similarly, after 7 days of immersion in alcohol-based or alcohol-free mouthwashes, the color variability among the same resin composites also remained similar (*p* = 0.113, *p* = 0.332, and *p* = 0.151, respectively) ( Table 3).

After 24 hours of immersion in an alcohol-based mouthwash, the Filtek Z350XT resin composite exhibited significantly greater color variation than the Opallis (*p* = 0.005) and Tetric N-Ceram (*p* = 0.003) resin composites. Likewise, after 7 days in the same mouthwash, Filtek Z350XT showed significantly greater color variation compared to both Opallis and Tetric N-Ceram (*p* = 0.027 and *p* = 0.004, respectively). In contrast, no significant differences in color variation were found among the three resin composites when immersed in an alcohol-free mouthwash, both at 24 hours (*p* = 0.057) and 7 days (*p* = 0.064) ( Table 4).

When comparing color variation between 24 hours and 7 days of immersion in alcohol-based and alcohol-free mouthwashes, only the Tetric N-Ceram resin composite immersed in an alcohol-based mouthwash demonstrated a significant increase in color variation over time (*p* = 0.036) ( Table 5).

## Discussion

This study evaluated the color stability of three resin composites immersed in mouthwashes, both with and without alcohol, over periods of 24 hours and 7 days. The findings led to a partial rejection of the null hypothesis, as only Tetric N-Ceram, when immersed in an alcohol-containing mouthwash, showed a significant increase in color change between the two time points.

Advances in nanofiller technology in dentistry have led to the development of nanohybrid resin composites, which combine the aesthetic properties of microfilled resin composites with the mechanical strength of hybrid materials [5]. These materials have been shown to enhance color harmony with dental tissues due to their so-called “chameleon effect” [5,22]. However, frequent use of mouthwash may adversely affect the surface properties of resin composites [12,13]. With regard to color stability, a ΔE value ≤ 3.3 is generally considered clinically acceptable [9,23-26]. All resin composites in this study exceeded the clinically acceptable ΔE threshold at every time point evaluated.

Notably, some studies did not report clinically significant color changes, which may be attributed to differences in methodology, including disc dimensions, photopolymerization times, the type of spectrophotometer used, and the formulation of the mouthwashes [23,27,28]. A systematic review found that in 10 out of 15 studies, exposure to mouthwashes did not result in noticeable color changes [7]. However, these findings could not be confirmed through meta-analysis due to methodological heterogeneity across the included studies [7]. The same review also indicated that color changes were more frequently observed when the daily use of mouthwash was simulated for one year or longer [20,24,30].

In the present study, the color variation observed in the resin composites Tetric N-Ceram, Filtek Z350XT, and Opallis was similar after immersion in both alcohol-based and alcohol-free mouthwashes. These findings are consistent with the results reported by Baig *et al*. [23], but contrast with those of Khosravi *et al*. [18] and Toz Akalin *et al*. [24]. This discrepancy may be attributed to differences in the types of resin composites used. For example, microhybrid resin composites, in contrast to nanohybrid ones, tend to exhibit greater color change due to their larger filler particles, which may facilitate the infiltration of staining agents at the matrix–filler interface [18,24,25].

Conversely, the Filtek Z350XT resin composite demonstrated significantly greater color variation compared to Opallis and Tetric N-Ceram when immersed in an alcohol-based mouthwash at both 24 hours and 7 days. This may be attributed to the lower filler content of the Filtek Z350XT resin composite, as it has been reported that resin composites with lower inorganic filler content undergo more color change due to the larger resin matrix volume, which allows for greater water sorption [25,26]. Consequently, some studies have indicated that color change in resin composites is related to the role of water, as water absorption can cause hygroscopic expansion in the resin network, leading to issues such as color change, degradation of the filler/matrix interface, reduced hardness, and loss of wear resistance [25,27,28]. Additionally, the presence of alcohol may compromise the surface integrity of resin composites, potentially contributing to color variation. Therefore, Listerine® Cool Mint mouthwash, with its lower pH due to the presence of benzoic acid and alcohol, can significantly accelerate the degradation of the resin composite structure over time. This is a complex process that results in the disintegration of the polymeric matrix and leads to significant issues, including filler–matrix separation and collapse, release of residual monomers, surface wear, and color variation [28,29]. This behavior could also be explained by the retention of solvents in microcracks induced by rapid polishing, a factor that remains underexplored in the literature.

It has been reported that the type of resin matrix also plays an important role in color variation [24,28]. Urethane dimethacrylate (UDMA) is recognized as more resistant to staining than bisphenol A-glycidyl methacrylate (Bis-GMA), due to its lower water absorption and solubility. Bis-GMA is a highly viscous monomer, which is why triethylene glycol dimethacrylate (TEGDMA) is typically added. TEGDMA contains an ethoxy group with high affinity for water molecules through hydrogen bonding with oxygen, leading to increased hydrophilicity of the resin composite [24,27,28]. All three resin composites used in this study contained these monomers. However, it is important to highlight that Filtek Z350XT, which has lower inorganic filler content [25,26], also contains higher proportions of Bis-GMA, TEGDMA, and UDMA compared to Tetric N-Ceram and Opallis. These differences in chemical composition may help explain the significant ΔE values observed after immersion in alcohol-based mouthwash at both 24 hours and 7 days.

When comparing color variation over time—between 24 hours and 7 days of immersion in an alcohol-based mouthwash—Tetric N-Ceram exhibited greater color change, possibly due to the presence of alternative photoinitiators specific to the brand, such as Ivocerin [3,16]. This, in combination with its larger filler particle size (40–3000 nm), may be less favorable over time [18,24,25], unlike the Filtek Z350XT and Opallis resin composites, which contain camphorquinone and have particle sizes ranging from 5–20 nm and 5–50 nm, respectively. Regarding photoinitiators, it has been suggested that incomplete polymerization may result in increased color change in resin composites [28,30]. However, since no measurements of double bond conversion or hardness were performed in this study, this explanation should be interpreted with caution. Therefore, it is crucial that formulations with alternative photoinitiators achieve a high degree of conversion, which can be effectively promoted using polywave light-emitting diode (LED) devices [31,32].

Listerine® Cool Mint contains alcohol and essential oils such as eucalyptol, thymol, menthol, and methyl salicylate, which can contribute to the degradation of resin composites and increase their surface roughness [27]. As a result, greater color variation was expected in composites immersed in this mouthwash compared to those exposed to an alcohol-free alternative, such as Listerine® Zero. However, the alcohol concentration might not have been sufficient to cause significant differences between the nanohybrid resins. Moreover, the uniform polishing protocol applied to all specimens may have minimized potential surface-level differences [3,33].

A strength of this study was the use of the CIE L*a*b* color system in combination with a digital spectrophotometer, which eliminates subjective variability in color perception and allows for consistent, objective, and accurate evaluation of color changes over time [9]. The CIE L*a*b* system provides a color space that more comprehensively reflects visual perception by incorporating luminance and chromatic dimensions, resulting in more reproducible measurements. Another strength was the assessment of color variation under medium- and long-term conditions (2 and 14 years of clinical use) using mouthwashes [11,12,17,20]. While most studies simulate aging with 24-hour continuous immersion—equivalent to approximately 2 years of clinical use at 2 minutes per day—this study also included a 7-day immersion, representing about 14 years of use under the same daily conditions [11,12,17].

The limitations of this study include the fact that the storage medium did not fully replicate the complexity of the oral environment. It is also important to acknowledge that color variation depends on multiple factors that are difficult to reproduce *in vitro*, such as saliva, oral microflora, salivary pellicle, pH fluctuations, diet, and beverages. Another limitation was the use of continuous immersion in mouthwashes, which does not accurately reflect clinical conditions. Continuous immersion protocols may overestimate color change compared to clinical use, which typically involves intermittent exposure (30 seconds, 2–3 times per day). Therefore, well-designed randomized clinical studies are needed to validate these findings under clinical conditions.

## Conclusions

In this *in vitro* study, the color variation in the composite resins Opallis, Tetric N-Ceram, and Filtek Z350XT was similar when comparing immersion in alcohol-based and alcohol-free mouthwashes, both at 24 hours and 7 days. However, Filtek Z350XT exhibited greater color change than the other resins when exposed to the alcohol-based mouthwash at both time points. No significant differences were observed between the resins when using the alcohol-free mouthwash. Additionally, only Tetric N-Ceram showed a significant increase in color variation over time in the presence of alcohol. It is noteworthy that all evaluated resins exceeded the clinically acceptable threshold for color variation at all immersion times, highlighting the potential chromatic impact of mouthwashes, particularly those containing alcohol.

## Figures and Tables

**Table 1:** Technical profile of the products used.

Product	Composition	Filler % (wt-vol)	Manufacture	Lot
Opallis A2	Matrix: Bis-GMA, Bis-EMA, UDMA, TEGDMA. Filler: The loads are a combination of silanized barium-aluminum silicate glass and nanoparticles of silicon dioxide, camphorquinone as photoinitiator, accelerators, stabilizers and pigments	79.8 wt - 58 vol	FGM, Santa Catarina, Brazil	220922
Tetric® N-Ceram A2	Matrix: Bis-GMA, Bis-EMA, UDMA Filler: Dimethacrylates, additives, catalysts, stabilizer sand pigments, barium glass, ytterbium trifluoride, mixed oxide and prepolymerized filler	81 wt - 57 vol	Ivoclar Vivadent, Schaan, Liechtenstein	Z062JG
Filtek Z350XT A2	Matrix: Bis-GMA, UDMA, TEGDMA, Bis-EMA Filler: zirconia/silica, barium glass, ytterbium trifluoride, mixed oxide prepolymer	75wt - 59.5 vol	3M, ESPE, St. Paul, MN, USA	9976007

**Table 2:** Table Mouthwashes Used.

Mouthwashes	Manufacture	Composition	pH
Listerine® Cool Mint	Johnson & Johnson Healthcare Prod.	Thymol, eucalyptol, methyl salicylate, menthol, water, sorbitol solution, alcohol (30%), poloxamer 407, benzoic acid, mint and mint essences, sodium saccharin, sodium benzoate and green dye 3.	4.35
Listerine® Zero	Johnson & Johnson Healthcare Prod.	Thymol, eucalyptol, methyl salicylate, menthol, water, sorbitol solution, poloxamer 407, benzoic acid, mint and peppermint essences, sodium saccharin, sodium benzoate and green dye 3.	6.02

**Table 3:** Comparison of the color variation (ΔE) of three different resin composites immersed for 24 hours and 7 days in alcohol-based and alcohol-free mouthwash.

Time	Resin composite	Alcohol	n	Mean	SD	SE	95% CI		p*	p**
							LL	UL		
24 hours	Opallis	Yes	15	3.70	1.18	0.31	3.04	4.35	0.259	0.219
		No	15	3.40	0.84	0.22	2.94	3.87	0.986	
	TNC	Yes	15	3.54	1.35	0.35	2.79	4.29	0.716	0.210
		No	15	3.88	0.89	0.23	3.39	4.38	0.231	
	Z350XT	Yes	15	5.62	1.76	0.45	4.65	6.60	0.052	0.197
		No	15	4.95	2.42	0.62	3.61	6.29	0.551	
7 days	Opallis	Yes	15	4.04	1.54	0.40	3.19	4.90	0.498	0.113
		No	15	3.46	0.96	0.25	2.93	3.99	0.986	
	TNC	Yes	15	3.77	1.08	0.28	3.17	4.37	0.579	0.332
		No	15	3.93	0.87	0.22	3.45	4.41	0.221	
	Z350XT	Yes	15	5.69	1.74	0.45	4.73	6.65	0.111	0.151
		No	15	4.94	2.17	0.56	3.74	6.14	0.246	

**Table 4:** Comparison of color variation (ΔE) between resin composites immersed for 24 hours and 7 days in alcohol-based and alcohol-free mouthwash.

Alcohol	Resin composite	24 hours					7 days				
		Mean	SD	p*	p**		Mean	SD	p*	p**	
					TNC	Z350XT				TNC	Z350XT
Yes	Opallis	3.70	1.18	0.002*	0.943	0.005**	4.04	1.54	0.005*	0.841	0.027**
	TNC	3.54	1.35			0.003**	3.77	1.08			0.004**
	Z350XT	5.62	1.76				5.69	1.74			
No	Opallis	3.40	0.84	0.057			3.46	0.96	0.064		
	TNC	3.88	0.89				3.93	0.87			
	Z350XT	4.95	2.42				4.94	2.17			

**Table 5:** Comparison of color variation (ΔE) of three resin composites between 24 hours and 7 days of immersion in alcohol-based and alcohol-free mouthwash.

Resin composite	Alcohol	f -i	SD	SE	95% CI		t	p*
					LL	UL		
Opallis	Yes	0.35	0.83	0.21	-0.11	0.81	1.62	0.063
	No	0.06	0.62	0.16	-0.28	0.41	0.39	0.350
TNC	Yes	0.23	0.45	0.12	-0.02	0.48	1.94	0.036*
	No	0.04	0.49	0.13	-0.23	0.31	0.33	0.373
Z350XT	Yes	0.07	0.64	0.16	-0.29	0.42	0.40	0.347
	No	-0.02	0.93	0.24	-0.53	0.50	-0.08	0.471

## Data Availability

The datasets used and/or analyzed during the current study are available from the corresponding author.
